# Features of the Nurse-Patient Relationship: Insights from a Qualitative Review Using Artificial Intelligence Interpretation

**DOI:** 10.3390/curroncol31120567

**Published:** 2024-12-02

**Authors:** Elsa Vitale, Luana Conte, Roberto Lupo, Stefano Botti, Annarita Fanizzi, Raffaella Massafra, Giorgio De Nunzio

**Affiliations:** 1 Scientific Directorate, IRCCS Istituto Tumori “Giovanni Paolo II”, Viale Orazio Flacco 65, 70124 Bari, Italy; 2 Department of Physics and Chemistry, University of Palermo, 90128 Palermo, Italy; luana.conte@unipa.it; 3 Advanced Data Analysis in Medicine (ADAM), Laboratory of Interdisciplinary Research Applied to Medicine (DReAM), University of Salento and ASL (Local Health Authority), 73100 Lecce, Italy; giorgio.denunzio@unisalento.it; 4 “San Giuseppe da Copertino” Hospital, ASL (Local Health Authority), 73100 Lecce, Italy; roberto.lupo@asl.lecce.it; 5 Hematology Unit, Oncology and Advanced Technologies Department, Azienda USL-IRCCS of Reggio Emilia, 42123 Reggio Emilia, Italy; stefano.botti@ausl.re.it; 6 Laboratorio di Biostatistica e Bioinformatica, IRCCS Istituto Tumori “Giovanni Paolo II”, 70124 Bari, Italy; a.fanizzi@oncologico.bari.it (A.F.); r.massafra@oncologico.bari.it (R.M.); 7 Laboratory of Biomedical Physics and Environment, Department of Mathematics and Physics “E. De Giorgi”, University of Salento, 73100 Lecce, Italy

**Keywords:** art, cancer, creativity, nursing, relationship

## Abstract

Introduction: This qualitative literature review explored the intersection of art, creativity, and the nurse–patient relationship in the context of oncology nursing. It delved into the perceptions and reflections of nurses as captured by Generative Artificial Intelligence (GAI) analysis from two specialized nursing databases. Methods: The protocol was registered on the Open Science Framework (OSF) Platform. A comprehensive search was conducted in CINAHL, the British Nursing Database, and the Nursing & Allied Health Database, using keywords related to art, cancer, creativity, nursing, and relationships. The extracted qualitative research studies were then analyzed using GAI to identify key themes and insights. Results: The analysis revealed profound considerations regarding the role of nurses in oncology and palliative patient care. Nurses acknowledged the spiritual dimension through religious and spiritual practices, while emphasizing authentic presence and empathic communication. They actively addressed patient concerns, adapted to challenges, and engaged in continuous professional development. The insights from the GAI interpretation underscored the significance of empathy, creativity, and artistry in nurturing meaningful nurse–patient connections. Conclusions: The GAI-enabled exploration provided valuable insights into several dimensions of care, emphasizing the importance of spiritual sensitivity, empathic communication, and ongoing professional growth. As technology and human care converge, integrating artistry into the nurse–patient relationship could enhance patient experiences, improve outcomes, and enrich the oncology nursing practice.

## 1. Introduction

The nature of oncology nursing requires extensive clinical expertise and dedication across all levels of the healthcare system involved in its processes [1]. Understanding the various aspects of this context and adopting effective approaches to address them is essential for the oncology nursing role [2]. The literature has described nursing as a form of “art therapy” due to its inherently supportive approach across all nursing tasks [3]. More specifically, oncology nursing has been considered an important support for cancer patients, helping them express their emotions and contributing to the creation of a secure and caring healthcare environment [2,3].

A cancer diagnosis and treatment undoubtedly induce significant stress, anxiety, and other emotional disorders [4]. Integrating art into nursing tasks can play an important role in helping nurses better understand their own emotions [5]. Furthermore, nursing care can help both nurses and patients understand diseases more comprehensively and enhance satisfaction with the care provided [6]. Oncology nursing delivers high-quality cancer care across the continuum of care. The Oncology Nursing Society has highlighted improvements in the oncology nursing workforce, attitudes, and experience in cancer care, as well as novel care delivery approaches in clinical practice [7]. In this context, oncology nursing care adopts a person-centered health policy [8], incorporating various nonpharmacological interventions [9] aimed at promoting health, self-worth, achievement, and social involvement [10]. These interventions benefit not only patients but also their caregivers, families, and healthcare professionals [11].

Several studies have explored the numerous positive effects of creative involvement in cancer patients, addressing their psycho-physical and spiritual dimensions [12,13,14]. A variety of art meditation programs, including art therapy and art-making activities have been implemented across different phases of caring, both in hospital and outpatient oncology settings [15,16,17,18]. In this regard, Watson’s theory [19] highlights the power of creative art expression interventions by integrating philosophy and science into four key sub-dimensions: human beings, health, environment, and nursing [20]. These sub-dimensions can be effectively incorporated into patient-centered care pathways, fostering hopeful attitudes to improve well-being perceptions, caring, and communication performances, which positively influence patients’ expressions [20,21].

According to the World Health Organization (WHO), spiritual well-being should be regarded as the fourth dimension of health, alongside physical, psychological, and social dimensions [22]. The spiritual dimension has been described as a state in which patients can autonomously manage their lives according to their personal purposes [23]. Among cancer patients, particularly younger individuals, higher levels of spiritual well-being can help them cope with mental health crises, including the possibility of dying [24,25].

Arts in cancer communities can help patients enhance their experiences and goals during emergency and rehabilitation phases, enabling them to better navigate intricate variations in healthcare solutions [25].

This qualitative literature review aimed to highlight the available evidence on the intersection of art, creativity, and the nurse–patient relationship in oncology nursing. To achieve this, we reviewed the literature to explore cancer nursing reflections on art, creativity, and the nurse–patient relationship in their practice, capturing these themes through Generative Artificial Intelligence (GAI) analysis from two specialized nursing databases.

## 2. Materials and Methods

### 2.1. Search Strategy

The protocol was registered on the Open Science Framework (OSF) Platform (https://doi.org/10.17605/OSF.IO/V7AC8 (accessed on 13 November 2024)). Research questions were developed using the “Population, Intervention, and Outcome” (PIO) approach (Table 1). Keywords based on MeSH terminology were as follows: “art”, “cancer”, “creativity”, “nursing” and “relationship”, combined with the Boolean operators AND/OR (Table 1) [26].

**Figure 1:**
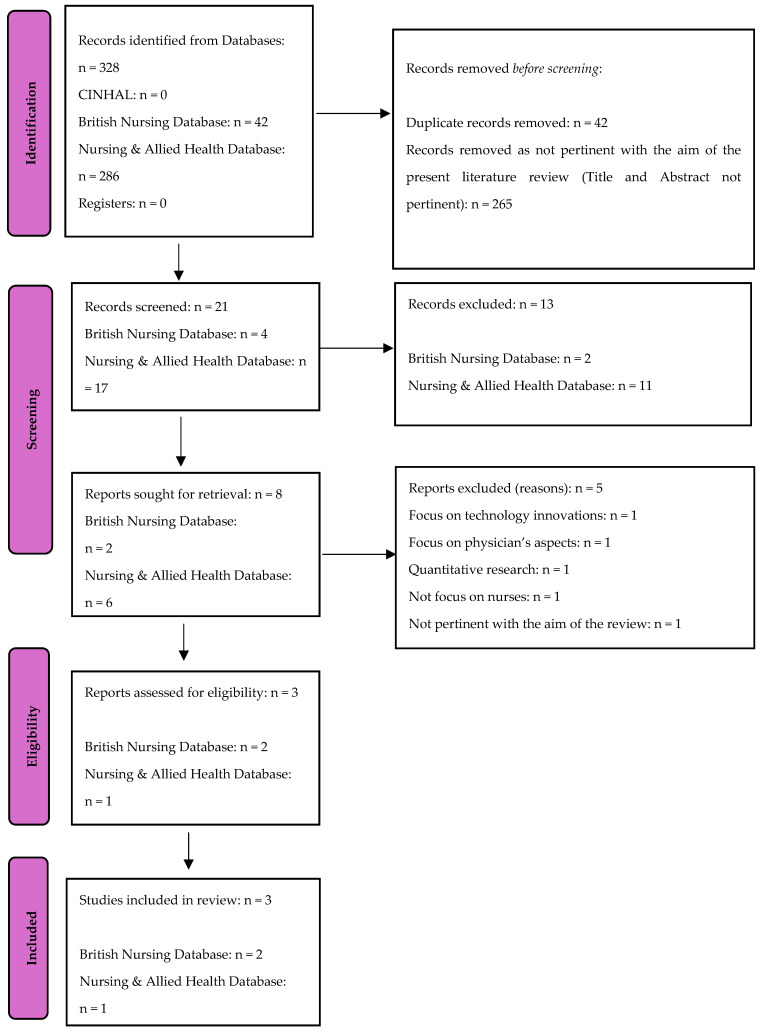
PRISMA 2020 flow diagram adopted in the review.

This qualitative literature review was conducted through the Preferred Reporting Items for Systematic Reviews and Meta-Analyses approach (PRISMA) [27] (Figure 1). CINAHL, the British Nursing Database, and Nursing & Allied Health Database were explored and only articles written in English were included in the review [26].

### 2.2. Study Selection

Titles and abstracts were read in their full-text versions to better assess the pertinence of each article with the aim of the review. Inclusion criteria embraced the following:

- Full-text availability;
- Qualitative research articles;
- Publication dates: from 1st January 2018 to 30 June 2023, to achieve the most recent highlights in this nursing theme;
- Authors were oncology/cancer nurses without any limitations to work experience in the oncology/cancer field;
- Published manuscript in English.

Qualitative research articles which did not meet the criteria or covered the following were excluded, specifically:

- Manuscripts focusing on other healthcare professionals;
- Quantitative research articles;
- Systematic literature reviews;
- Manuscripts published in languages other than English;
- Medical research frameworks.

Initially, records were identified through a systematic database search and uploaded to reference management software, where duplicate studies were removed (n = 42). Then, two independent reviewers (E.V. and L.C.) assessed the title and abstract of the identified studies for inclusion, and unsuitable reports were removed. After that, articles were made available, and the full text was assessed more closely for eligibility. Disagreements about whether a study should be included were resolved through discussion and mutual agreement. If the disagreement persisted, a third reviewer (R.L.) acted as an arbitrator.

### 2.3. Selected Records

During the first phase of this systematic review and meta-analysis, a total of 328 records were identified. Among these, 42 records were removed as duplicates and 265 were removed as titles and abstracts did not cover the aim of this qualitative review. After screening, a total of 21 potential records were obtained. However, 13 first and, subsequently, other 5 manuscripts, were excluded as they did not meet the inclusion criteria after reviewing their methodology section. Finally, the remaining 3 manuscripts were selected for inclusion in this qualitative review, as shown in Figure 1.

### 2.4. Study Quality Assessment

The three manuscripts selected for this qualitative review were assessed in their quality thanks to the “Consolidated criteria for reporting qualitative research (COREQ)” checklist [27]. For each requirement, a brief answer was indicated to better assess the quality of the manuscripts selected for this review (Table 2).

### 2.5. Data Analysis

The extracted qualitative research studies were analyzed through Generative AI (GAI) to identify key themes and insights. The analysis was conducted using ChatGPT (4.0), a large language model, to identify key themes, insights, and reflections present in the selected studies. Prompts were designed to facilitate the extraction of core themes from the analyzed databases. The GAI model was supplied with specific excerpts from the studies instead of the full articles, enabling it to concentrate on relevant content while preserving the context of the findings. This method enabled a consistent and objective synthesis of insights across different studies, which would be more time-consuming and potentially subjective through a manual narrative approach [31].

## 3. Results

The obtained data highlighted nurses’ reflections from the two databases, underscoring the importance of empathy, authentic presence, and empathic communication in oncology and palliative patient care. Structural and personal challenges affected the implementation of the spiritual dimension of care. Learning from experienced colleagues was essential for developing communication competencies and adapting to the complexities of the oncology environment (Table 3). Data suggested overlapping between art, creativity, and the nurse–patient relationship and empathy, authentic presence, and empathic communication in oncology and palliative patient care (Table 3).

Five comprehensive sub-dimensions were identified to better describe the nursing role in both oncology and palliative care and their related insights (Table 4), specifically:

1. Spiritual Dimension of Care in Palliative Patients;
2. Authentic Presence and Empathic Communication;
3. Management of Patient Concerns;
4. Education and Professional Development;
5. Challenges and Practical Aspects.

## 4. Discussion

The GAI-assisted analysis provided valuable insights into the multifaceted dimensions of care, emphasizing the importance of spiritual sensitivity, empathic communication, and ongoing professional growth. As technology and human care converge, integrating artistry into the nurse–patient relationship could enhance patient experiences, improve outcomes, and enrich the oncology nursing practice. Five key sub-dimensions have been found in this qualitative review.

### 4.1. Spiritual Dimension of Care in Palliative Patients

Nurses encourage the spiritual aspect of care, associating religious and spiritual aspects and considering patients’ opinions, thanks to the recognition of the spiritual dimension through structural and professional support. Spirituality represents a multidimensional perception which introduces a double meaning, as life and transcendence [32,33,34], between an intra- and an inter-personal dimension [35] and searches the essential meaning of life linked to religion, art, music, and nature [36]. To alleviate patients’ suffering and enhance their quality-of-life perceptions, healthcare professionals and nurses could benefit from knowledge about spiritual need assessments among cancer patients, enabling them to address specific care requirements effectively [37]. However, evidence suggested the presence of possible barriers to promoting effective spiritual support. A common univocal definition of the term “spirituality” [35] is associated with the scarce availability of time [35,38,39], privacy [40], and economic, personal, and cultural aspects combined with the need for professional and educational development in this matter [35,38].

### 4.2. Authentic Presence and Empathic Communication

Nurses encourage authentic presence with devotion and openness by encouraging empathic communication and awareness strategies [41], which are recognized as essential in improving patients’ care in all nursing fields, such as prevention, treatment, rehabilitation, education, and health promotion [42,43]. Effective communication can positively influence patient caring independence and satisfaction [10,44,45,46], defending patients from adverse health effects [47,48] and, thanks to humanistic and unique behaviors, a serene atmosphere was provided reducing the patient’s worry and encouraging them.

According to some researchers, empathy is one of the communication instruments adopted to understand others [49] and constitutes an important function in effective nurse–patient communication [41]. Evidence suggested empathetic nurse–cancer patient communication, introducing an empathetic communication model [42] also associated with a non-verbal approach [50] to elicit a patient’s empathic opportunity and share the patient’s emotion/experience and promote coping, which seemed to be associated with the social support [42], also suggesting a strategy to solve the problem and perform a positive action to their resolution [51].

Emphatic communication requires listening, providing information, encouraging, ensuring that they look after themselves, helping them cope with contextual barriers, and sharing personal thoughts [52].

### 4.3. Management of Patient Concerns

Nurses address both the physical and psychological patients’ dimensions through nurse–patient communication at both admission and discharge. The literature suggests that interventions could better address patient’s needs by integrating them into existing contexts within the national health system. These contexts include management approaches, organizational culture, and the dynamics of power in relationships between patients and healthcare professionals. Such integration aims to create a model which effectively connects the various actors within the healthcare system.

Effective interventions to address patient difficulties should be as comprehensive as possible, aiming to better integrate information, and relationships between patients, professionals, and bureaucracies, while mitigating limitations such as restricted interventional contexts, insufficient system dissemination strategies, and inadequate empirical evaluations in staff management [53,54].

### 4.4. Education and Professional Development

Oncology nurses require ongoing knowledge which positively influences younger colleagues and promotes empathic communication through continuous education [55]. Nursing training includes both practical and theoretical strategies [56] that provide nurses with sufficient knowledge, attitudes, and competencies to improve public health [57]. Nursing education is a mixture of theory and practice to achieve the knowledge, competencies, and attitudes in nursing care [58]. However, nursing education provides a complicated and huge number of demands, which has not yet been reached [59].

### 4.5. Challenges and Practical Aspects

Nurses experience a lot of time with patients and their illnesses [60], influencing patient perceptions [61]. Nurses may provide balanced nursing services combined with additional performances, such as case management and practice leadership, nursing advocacy, and illness prevention [62], requesting effective communication, flexibility, and creativity. Nurses should be prepared with sufficient skills by assessing requirements for the requested workforce in the work setting [63].

Research has shown that the nursing work context is a fundamental factor in a patient’s perception of quality. In fact, when patients have positive experiences with nursing care, nurses also work in a good and healthy environment [64,65], which helps them to reach both their own and organizational goals [66]. To identify key factors in nursing practice, cost-effectiveness, transparency, and patient-centered care policy, nurses should be able to make decisions to improve patient care, by linking several policies requiring the participation of both nurses and nursing management [67]. Nurses need to be empowered to create their own environment with a strong nursing practice [68], which will lead to more positive patient outcomes [69].

### 4.6. Strengths and Limitations

This literature review was conceived as an intersection between arts and creativity in nursing. Due to its specific focus, the authors decided to consider only the three specialized nursing databases for their research. However, the results revealed a multidisciplinary involvement of the nursing profession in arts and creativity associated with the spiritual dimension, emphatic communication, management, education, and challenges in clinical practice. This multidisciplinary involvement could be seen as integral to the essence of the nursing profession, which should not be considered in isolation, but as a profession that inevitably intersects with other disciplines. Nursing in this sense is concerned with caring for the human being in a holistic manner, recognizing that the individual is not a separate entity from their surrounding environment [10,13].

## 5. Conclusions

The GAI-enabled exploration provides valuable insights into the multifaceted dimensions of care, emphasizing the importance of spiritual sensitivity, empathic communication, and ongoing professional growth. As technology and human care converge, integrating artistry into the nurse–patient relationship can enhance patient experiences, improve outcomes, and enrich the practice of oncology nursing.

Natural Language Processing (NLP) techniques, such as sentiment and emotion analysis [31] could allow some insights into the emotions of interviewers. Traditional NLP approaches typically identify basic emotions like positive, negative, or neutral which could detect emotions, like joy, anger, or sadness, missing the richness of language found in open-ended responses. On the other hand, the recent literature suggests new models to process questionnaires to highlight what participants say in their clinical practice [32].

The development of AI-driven Large Language Models (LLMs) [17] such as OpenAI’s GPT and ChatGPT, have transformed the field of NLP, allowing for a deeper comprehension of human expression in extensive datasets, and the capability to grasp context, discern subtle shifts in tone, and produce coherent, contextually relevant outputs.

## Figures and Tables

**Table 1:** The “Population, Intervention, and Outcome” (PIO) approach to the qualitative literature review.

Population	Oncology/Cancer Nurse(s)
Intervention	Intersection of art, creativity, and the nurse–patient relationship
Outcomes	Perceptions and reflections of nurses as captured by Generative Artificial Intelligence (GAI) analysis from two specialized nursing databases.

**Table 2:** Consolidated criteria for reporting qualitative studies (COREQ): a 32-item checklist.

COREQ-Items	Author(s); Publication Year		
	Evangelista et al., 2021 [28]	Guevara Lozano et al., 2019 [29]	Chan et al., 2019 [30]
Domain 1: Research team and reflexivity			
Personal Characteristics			
1. Interviewer/facilitator	Not specified	Focus group	Focus groups and individual interviews
2. Credentials	Not specified	Not specified	Not specified
3. Occupation	Not specified	Nursing group was assigned for two months to follow the movements of randomly selected patients and their family caregivers	Hospital-based cancer nurses in Hong Kong
4. Gender	Not specified	Not specified	Not specified
5. Experience and training	Not specified	Not specified	A bachelor’s degree in nursing and work experience in oncology up to 3 years
Relationship with participants			
6. Relationship established	Not specified	Not specified	There were no pre-existing relationships
7. Participant knowledge of the interviewer	The interviews were carried out to clarify any doubts of participants	Identification and description of the institutional route of patients and their family caregivers and the encounter with nursing	They were purposively sampled and recruited by the research assistant (RA)
8. Interviewer characteristics	Among the nursing theories that address the spiritual dimension in patient care, the Theory of Human Caring stands out	Admission, general assessment and pain permanent, care of basic needs, change in shift, administration of medications, and discharge	The criteria for the inclusion of nurses were those with at least two years of nursing experience and one year in the current oncology work setting
Domain 2: study design			
Theoretical framework			
9. Methodological orientation and Theory	The sample quantum was given for convenience and was completed in view of the saturation of the interviews.	Not specified	Personal reflexivity and epistemological reflexivity
Participant selection			
10. Sampling	Convenience	Consecutive	Purposively sampled and recruited
11. Method of approach	Face to face	Face to face	Face to face
12. Sample size	10, 9 females, 1 male; aged 33–60 years	Not specified	11 hospital-based cancer nurses in Hong Kong
13. Non-participation	Professionals removed from their activities, due to vacation or leave, and those who did not work with a patient in palliative care	Not specified	Not specified
Settings			
14. Setting of data collection	Hospital	Clinic	Hospital
15. Presence of non-participants	Not specified	Not specified	Not specified
16. Description of sample	Nurses with at least six months of experience in the selected institution and worked with a patient in palliative care	Not specified	Nurses with at least two years of nursing experience and one year in the current oncology work setting
Data collection			
17. Interview guide	The empirical material began to show redundancy and repetition from the interviewer and the analysis of the empirical material (Laurence Bardin)	The process implied: Identification and description of the institutional route of patients and their family caregivers with Nursing; Complementing the aforementioned; Encounters with patients and their family caregivers within their care route; Upon identifying the encounters among patients and their family caregivers and nurses.	Two focus group interviews, which consisted of four to five participants per group, were conducted by a skilled moderator and a note taker
18. Repeat interviews	Not applicable	Not applicable	Not applicable
19. Audio/visual recording	Not applicable	Another group of observers analyzed the typical day for nurses	Not applicable
20. Field notes	Not applicable	Some decisive moments were expressed during the care process and continued until the moment of discharge	Focus group
21. Duration	August–December, 2019	2016–2018	2 July 2017–2 January 2018
22. Data saturation	The sample quantum was given for convenience and completed with the saturation of the interviews	The sample reached for the period considered	Interviews were conducted until thematic data saturation was reached after the interviews with 11 participants, where no new/additional ideas were identified in the data
23. Transcripts returned	Not applicable	Not applicable	Not applicable
Domain 3: analysis and findings			
Data analysis			
24. Number of data coders	N1, N2, N3 and so on	Not specified	Not specified
25. Description of the coding tree	The anonymity of the participants, they were identified using the letter N (nurse), followed by the ordinal number	Not specified	
26. Derivation of themes	Themes derived from the data	The type and form of communication, knowledge, tone of the relationship, the exercise of roles, and guarantee of necessary resources, including the pedagogic methods and the way of assessing care	Themes derived from the data
27. Software	Not applicable	Not applicable	Not applicable
28. Participant checking	Not applicable	Not applicable	Not applicable
Reporting			
29. Quotations presented	Not applicable	Themes were discussed	Themes were discussed
30. Data and findings consistent	The data presented and the findings were consistent	The data presented and the findings were consistent	The data presented and the findings were consistent
31. Clarity of major themes	Major themes were presented in the findings	Major themes were presented in the findings	Major themes were presented in the findings
32. Clarity of minor themes	Minor themes were described	Minor themes were described	Minor themes were described

**Table 3:** GAI insights from nurses’ reflections in oncology and palliative patient care: analysis from two different databases.

**British Nursing Database**		
**Author(s); Publication Year**	**Aim**	**Reflections**
Evangelista et al., 2021 [28]	To transform significant moments in nurse–patient and family interactions, prioritizing “authentic presence” to enhance adaptation to illness.	Nurses explore “authentic presence” involving openness, unconditional love, and patient-focused care. This presence is fundamental to empathic communication and requires continuous training and learning from experienced colleagues.
Guevara-Lozano et al., 2019 [29]	How oncology nurses address communication and psychosocial needs in crowded wards.	Empathic communication eases patient concerns through explanations and reassurances, despite challenges in busy settings. Learning from senior nurses plays a pivotal role in enhancing communication skills.
**Nursing & Allied Health Database**		
**Author(s); Publication Year**	**Aim**	**Reflections**
Chan et al., 2019 [30]	How oncology nurses address communication with patients and psychosocial needs in crowded wards.	Empathic communication alleviates patient concerns through explanations and reassurances, though it remains a challenge in the hectic context. Training and learning from the experiences of senior nurses play a crucial role in refining communication skills.

**Table 4:** Comprehensive insights into nurse roles in oncology and palliative patient care from the GAI across combined databases.

Nursing Dimensions	Insights
Spiritual Dimension of Care in Palliative Patients	The spiritual dimension of care integrates religious and spiritual practices. Nurses respect and encourage patients’ beliefs. Structural challenges and professional training influence the realization of the spiritual dimension.
2. Authentic Presence and Empathic Communication	Nurses emphasize “authentic presence”, including devotion and openness. Empathic communication and responsiveness are vital. Empathy enhances nurse–patient communication.
3. Management of Patient Concerns	Nurses actively alleviate physical and psychological concerns. Communication during admission and discharge establishes connections.
4. Education and Professional Development	Learning from experienced nurses positively impacts younger colleagues. Adapting to oncology requires continuous education. Consistent practice enhances empathic communication.
5. Challenges and Practical Aspects	Balancing patient’s emotional needs with workload management is a daily challenge for nurses. Balancing patient’s needs with workload is a daily challenge. Patience and composure are vital. Effective communication requires flexibility and creativity.

## Data Availability

Data sharing is not applicable to our article.
